# Impact of Plant-Associated Bacteria on the In Vitro Growth and Pathogenic Resistance against *Phellinus tremulae* of Different Aspen (*Populus*) Genotypes

**DOI:** 10.3390/microorganisms9091901

**Published:** 2021-09-07

**Authors:** Greta Striganavičiūtė, Jonas Žiauka, Vaida Sirgedaitė-Šėžienė, Dorotėja Vaitiekūnaitė

**Affiliations:** Laboratory of Forest Plant Biotechnology, Institute of Forestry, Lithuanian Research Centre for Agriculture and Forestry, Liepų str. 1, Girionys, LT-53101 Kaunas, Lithuania; gretastrig6@gmail.com (G.S.); jonas.ziauka@lammc.lt (J.Ž.); doroteja.vaitiekunaite@lammc.lt (D.V.)

**Keywords:** *Pseudomonas*, *Paenibacillus*, *Populus tremula*, *Populus tremuloides*, secondary metabolites, *Phellinus tremulae*, hybrid poplar, phenols, flavonoids, carotenoids, chlorophyll

## Abstract

Aspens (*Populus tremula* and its hybrids), economically and ecologically important fast-growing trees, are often damaged by *Phellinus tremulae*, a rot-causing fungus. Plant-associated bacteria can be used to increase plant growth and resistance; however, no systematic studies relating the activity of symbiotic bacteria to aspen resistance against *Phellinus tremulae* have been conducted so far. The present pioneer study investigated the responses of two *Populus tremula* and two *P. tremula* × *P. tremuloides* genotypes to in vitro inoculations with, first, either *Pseudomonas* sp. or *Paenibacillus* sp. bacteria (isolated originally from hybrid aspen tissue cultures and being most closely related to *Pseudomonas oryzihabitans* and *Paenibacillus tundrae*, respectively) and, in the subsequent stage, with *Phellinus tremulae*. Both morphological parameters of in vitro-grown plants and biochemical content of their leaves, including photosynthesis pigments and secondary metabolites, were analyzed. It was found that both *Populus tremula* × *P. tremuloides* genotypes, whose development in vitro was significantly damaged by *Phellinus tremulae*, were characterized by certain responses to the studied bacteria: decreased shoot development by both *Paenibacillus* sp. and *Pseudomonas* sp. and increased phenol content by *Pseudomonas* sp. In turn, these responses were lacking in both *Populus tremula* genotypes that showed in vitro resistance to the fungus. Moreover, these genotypes showed positive long-term growth responses to bacterial inoculation, even synergistic with the subsequent fungal inoculation. Hence, the studied bacteria were demonstrated as a potential tool for the improved in vitro propagation of fungus-resistant aspen genotypes.

## 1. Introduction

Forest trees and their wood quality are threatened by pathogenic fungi [1,2]; thus, one of the most pressing problems in forestry, today, is the selection of tree genotypes for increased growth and better resistance to phytopathogens [3,4]. Therefore, more attention has recently been paid to potentially beneficial interactions between symbiotic bacteria and woody plants.

Endophytic bacteria can promote resistance to stress and pathogens, at the same time stimulating nutritional processes in their hosts, hence affecting overall growth [5,6,7,8]. Plant microorganisms can benefit their hosts through a variety of mechanisms, including biological nitrogen fixation and increased bioavailability of phosphorus (P), iron (Fe) and other mineral nutrients, as well as production of plant hormones and various antimicrobial biocontrol compounds [7,9,10,11]. Plant growth promoting bacteria are being more widely used in agriculture, as an eco-friendly alternative to chemical fertilizers and pesticides [7,12]; however, the potential application of such beneficial bacteria in forestry has not yet been sufficiently explored [13].

Tree resistance to pathogens is related to the general viability of trees and their ability to synthesize and mobilize secondary metabolites (SMs) [4,14,15,16,17]. Modern technologies for the improvement of forest tree planting material include quantitation of phenolic compounds, since these SMs perform numerous protective functions and their amount is related to general or specific plant pathogenic resistance [4,14,18,19]. Endophytic bacteria were proven to have an effect on plants’ secondary metabolism [16,20,21]; however, overall, little is known about the mechanisms of how this happens [16]. Moreover, studies show that the ability of different plant genotypes to synthesize SMs and mobilize them against pathogens is variable; therefore, genetic selection is appropriate in this respect [4,14,15,18].

Eurasian aspen (*Populus tremula* L.) are common pioneer trees in the Northern Hemisphere [15,22,23,24,25,26]. Furthermore, aspen and its hybrids are extensively grown for various commercial enterprises [14,15,25,27,28], as well as being used as a model species for not only woody plant experiments [28], but also plant–microbe interaction studies [29]. Even so, aspen trees suffer from fungal pathogens throughout their habitat range [15,30,31].

According to the data of the State Forest Service, aspens are the most vulnerable in Lithuanian forests. Data show that, in 2020, 13% of the total forest trees was damaged by diseases and 47.7% of them suffered from pathogen—*Phellinus tremulae* [32]. *P. tremulae* is a pathogenic fungus that is associated with aspen rot [14,33]. Several authors have identified it as one of the main pathogens affecting this genus [23,24,33,34,35,36], with some stating that up to 39% of trees may be infected in some areas [22], causing significant yield losses [34,35]. Despite the widespread nature of this pathogenic fungus, little research has been conducted on the antagonistic effects other microorganisms may have on this species. So far as we were able to determine, no systematic studies concerning the impact of different symbiotic bacteria on the formation of pathogenic resistance via SM production in *Populus* sp. have been conducted yet.

This paper highlights the ability of symbiotic bacteria belonging to the genera *Paenibacillus* and *Pseudomonas* to regulate morphogenetic processes and activate the synthesis of protective SMs in different aspen genotypes against phytopathogen *P. tremulae*. This pioneer study evaluates the possibilities of using plant growth promoting bacteria to improve growth, quality and resistance of forest planting material.

## 2. Materials and Methods

### 2.1. Populus Genotypes

Two aspen, *Populus tremula* (A-37 and R-38), and two hybrid aspen, *P. tremula × P. tremuloides* (174/10 and Wa13), genotypes were selected for the study. These genotypes were established in the laboratory of LRCAF Institute of Forestry (Lithuania) in 2017 and kept in vitro for two years via a series of bimonthly subcultures, before being used for the experiments described in this study.

### 2.2. Microorganisms

Two different strains of bacteria were used. Both were isolated from aspen in vitro cultures, that showed no initial external bacterial growth. Bacterial colonies were observed at the base of the microshoots, as well as on the surface of the medium surrounding the microshoot stems, after several bimonthly aseptic transfers. They appeared to be causing no harm to the plants; thus, based on their origin, were determined to be potentially endophytic. Subsequently, samples were taken for genetic testing. DNA extraction and, later, *16S rRNA* gene sequencing (universal 27F/800R–518F/1492R primer set) were conducted at the Macrogen sequencing center, Amsterdam, The Netherlands.

In vitro cultures of fungus *Phellinus tremulae* were established in LRCAF Forest Institute, using the samples from naturally infected *Populus tremula* trees in Lithuania and following the isolation procedure described by Holmer et al. [37].

### 2.3. Media

Plant cultures before and during the experiment were grown on solid Murashige and Skoog (MS) nutrient medium including vitamins, containing 20 g L^−1^ of sucrose and 4 g L^−1^ of gelrite (all the components were purchased from Duchefa Biochemie, Haarlem, The Netherlands). In both the pre-experimental and experimental treatments, the pH value of the medium for *Populus* shoot cultures was set at 5.8 before autoclaving for 30 min at 121 °C. For the experiment, 5.25 mL of medium was poured into each glass tube (20 × 150 mm).

Both species of bacteria were grown on a solid low salt Lysogeny broth (LB) medium (Duchefa Biochemie, The Netherlands). The pH value of the medium was set at 6.7 before autoclaving.

Fungus *P. tremulae* was grown on a solid malt medium containing 20 g L^−1^ of maltose, 8 g L^−1^ of yeast extract, 6 g L^−1^ of tryptone, 20 g L^−1^ of glucose and 6 g L^−1^ of gelrite (components purchased from Duchefa Biochemie). The pH value of the medium was set at 5.8 before autoclaving.

### 2.4. Inoculation of Plant Nutrient Medium with Bacteria and Fungi

For the first-stage inoculation, a day before the transfer of aspen explants, the MS medium in the tubes was spot-inoculated with isolate PP or with isolate L191B using an inoculation needle with ~1.4 × 10^6^ cfu (colony forming units) of PP or ~6.4 × 10^6^ cfu of L191B. The bacteria used were taken from bacterial colonies formed on the surface of solid LB medium from overnight cultures.

Bacterial concentrations were determined in a separate experiment using the serial dilution technique.

Fungus *P. tremulae* was used for the second-stage inoculation. The application of fungal inoculate basically followed the method that Seppänen et al. [38] previously used for the wood decay test; a 7 mm diameter sample from the edges of the mycelial growth was collected using a cork borer, transferred to a tube with the plant medium and placed in the middle, before the transfer of aspen explants.

The survival of both bacteria and fungus were determined visually.

### 2.5. Explants in Bacteria/Fungus Inoculated Medium

Apical segments of in vitro-developed shoots, 10 mm in length, with leaves removed, were used as explants. The explants were planted in individual tubes with different experimental variants—control (uninoculated medium) and medium inoculated with isolates PP of L191B. In the first culture stage, 30 explants were used in the experiment for each variant for genotype A-37, 34 explants for genotype R-38 and 39 explants for genotypes 174/10 and Wa13. Plants were kept in a growth chamber for six weeks. The temperature in the growth chamber was kept at 25/20 °C under a 16/8 h photoperiod (white light, irradiance 30 μmol m^−2^ s^−2^).

After six weeks, the morphological parameters (shoot length, largest leaf width) of in vitro-grown plants were recorded and the leaves were harvested for biochemical studies. Then, 10-mm-long apical shoots were transferred to fungus-inoculated tubes for the second-stage trials. Plants were grown for 6 additional weeks under the same conditions as mentioned before. Afterwards, plant vegetative growth parameters (rooting, shoot length and largest leaf width) were measured and leaves were collected for biochemical studies.

The general scheme of the conducted experiments is shown in Table 1.

### 2.6. Extract Preparation for Secondary Metabolite Analysis

Fresh leaf (500 mg) samples were stored at −20 °C until bioactive substances were analyzed. Before phenolic compound and flavonoid analysis, the samples were homogenized by mortar and pestle. Then, the homogenized material was shaken with 10 mL of 75% methanol for 24 h at 25 °C using a Kuhner Shaker X electronic shaker (Adolf Kühner AG, Birsfelden, Switzerland) at 150 rpm. Afterwards, the extracts were filtered through Whatman no. 1 filter paper, with retention of 5–8 µm.

### 2.7. Quantification of Total Phenolic Compounds

The total phenolic content was determined using Folin–Ciocalteu reagent according to Singleton et al. [39]. Leaf extracts (0.1 mL) were mixed with 0.1 mL of Folin–Ciocalteu reagent (2 N) and 2.5 mL of distilled water (dH_2_O). After 6 min, 0.5 mL of 20% (*w*/*v*) sodium carbonate was added into the mixture. The mixtures were left for 30 min at room temperature. The absorbance was measured using a Synergy HT Multi-Mode Microplate Reader (BioTek Instruments, Inc., Bad Friedrichshall, Germany) at 760 nm against the reagent blank (75% methanol). The phenol content was expressed as chlorogenic acid per gram of fresh weight of leaves (mg CAE/g). The standard calibration curve equation was y = 5.5358x − 0.0423 (R^2^ = 0.9975).

### 2.8. Quantification of Total Flavonoid Content

The flavonoid content in the extracts was determined according to a method described by Dróżdż et al. [40]. The extract solution (1 mL) was mixed with 0.3 mL of 5% (*w*/*v*) NaNO_2_ and, after 5 min, 0.5 mL of 2% (*w*/*v*) AlCl_3_ was added. After 6 min, the sample was neutralized with 0.5 mL of NaOH (1 M). The absorbance of the mixture was recorded at 470 nm on the Synergy HT Multi-Mode Microplate Reader. The flavonoid content was expressed in milligrams of catechin per gram of fresh weight of leaves (mg CE/g). The standard calibration curve equation was y = 11.616x + 0.0634 (R^2^ = 0.9983).

### 2.9. Quantification of Photosynthesis Pigments

Fresh leaves (0.2 g) were ground in acetone (VWR International, Fontenay-sous-Bois, France), then filtered through Whatman no. 1 filter paper, with retention of 5–8 µm. All pigment analyses were determined using a UV-VIS spectrophotometer 80+ (PG Instruments, Leicestershire, UK) at the following wavelengths: chlorophyll a, 662 nm; chlorophyll b, 644 nm; carotenoids, 440.5 nm. For the calculation of pigment content, the following models were used [41]:

(1) $$\mathrm{chl}\;a=9.784\times D_{662}-0.990\times D_{644}\frac{c\times V}{P\times 1000}$$

(2) $$\mathrm{chl}\;b=21.426\times D_{644}-4.650\times D_{662}\frac{c\times V}{P\times 1000}$$

(3) $$\mathrm{Carotenoids}=4.695\times D_{440.5}-0.268\times \left(\mathrm{chl}\;a+b\right)\frac{c\times V}{P\times 1000}$$

where *c* = pigment content (mg g^−1^), *V* = extract volume (mL) and *P* = fresh leaf material (g).

### 2.10. Statistical Data Analysis

The results presented in this study include the following parameters: rooting, shoot length, leaf width, chlorophyll a/b ratio, carotenoid content, total phenolic content and total flavonoid content. For the comparison of the obtained results, a two-tailed Welch’s *t*-test intended to compare samples with possibly unequal variances was performed to calculate the probability that the means of the different variants are equal [42] (Microsoft Excel).

## 3. Results

### 3.1. Isolation and Identification

**Figure 1:**
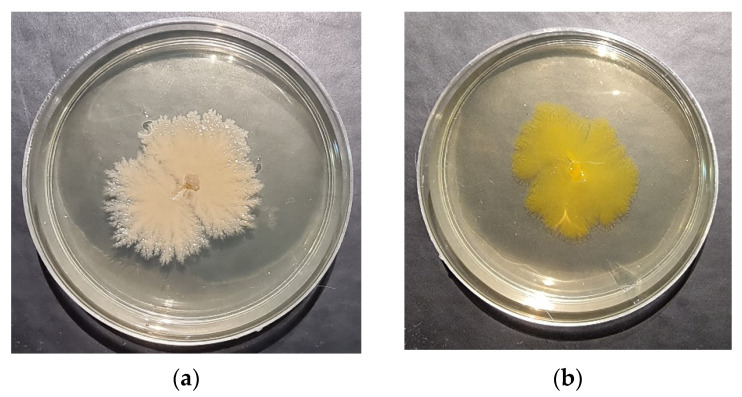
Morphology of bacterial species used in the experiments (on LB agar after 1 month incubation): (**a**) isolate PP—*Paenibacillus* sp.; (**b**) isolate L191B—*Pseudomonas* sp.

Isolate PP was isolated from in vitro cultures of hybrid aspen (*Populus tremuloides* × *P. tremula*; 51DF1001) grown in the laboratory of LRCAF Forest Institute, Lithuania. Based on *16S rRNA* gene fragment (1496 bp) sequencing, isolate PP was determined to belong to the *Paenibacillus* genus (Figure 1a). It was genetically close to *Paenibacillus tundrae* (99.06% Identity, NR_044525.1). The other bacterium used in the study (L191B) was isolated from the in vitro cultures of *Populus* hybrids L191 and 174/10 (*Populus tremula* × *Populus tremuloides*), established in the Forest Research Institute, Department of Forestry and Forest Tree Genetics, Poland (Figure 1b). This bacterium was assigned to the genus *Pseudomonas* (highly homologous with *Pseudomonas oryzihabitans* (fragment length, 1476 bp; 99.46% Identity, NR_117269.1)).

**Figure 2:**
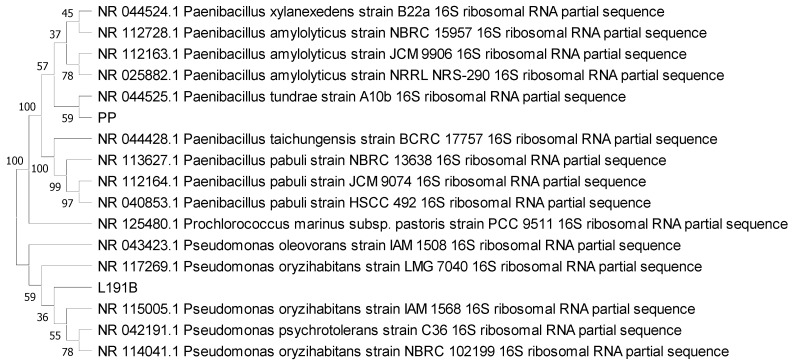
Phylogeny tree based on the neighbor joining method [44] using *16S rRNA* gene partial sequences of bacteria from this study (L191B and PP), as well as bacteria that were genetically closest (≥97% Identity) and cyanobacteria *Prochlorococcus marinus* as an outgroup. Branch length is 0.29527974. Bootstrap values (%) after 1000 iterations are shown next to the branches [45].

A phylogenetic tree with both bacteria and their closest genetic matches (≥97% Identity) based on sequences from the NCBI database is presented in Figure 2 (MEGA X) [43].

**Figure 3:**
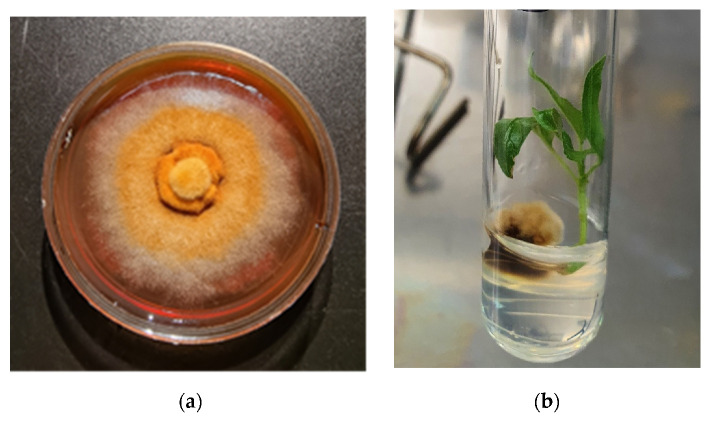
Fungus *P. tremulae*: (**a**) in a pure culture on malt nutrient medium; (**b**) with a *Populus* microshoot post inoculation.

Fungus *P. tremulae* was isolated from naturally infected *Populus tremula* trees in Lithuania and cultured on nutrient medium in LRCAF Forest Institute (Figure 3).

### 3.2. Effect of the Studied Microorganisms on Rooting of Different Populus Genotypes

For practical purposes, percentage of rooted explants can be taken as the main indicator of the general viability of *Populus* shoot cultures. Inoculation of plant nutrient medium with the studied microorganisms resulted in different genotype-specific outcomes, regarding the percentage of rooted explants in the four investigated *Populus* genotypes (Table 2).

The results, given in Table 2, show that, considering the percentage of rooting, only two of the four investigated *Populus* genotypes were affected positively by some of the studied microorganisms. Interestingly, both of these genotypes were aspens (*P. tremula*), in contrast to hybrid aspens (*P. tremula* × *P. tremuloides*). Genotype A-37 showed a positive response to the first-stage treatment with bacterium *Pseudomonas* sp. (average increase of up to 106%) and genotype R-38 showed a positive response to the second-stage treatment with fungus *Phellinus tremulae* (increase of up to 129%). It can be also noted that the first-stage treatment of R-38 explants with *Paenibacillus* sp., in contrast to *Pseudomonas* sp., prevented the positive effect of *P. tremulae* on the rooting of R-38 in the second stage. Meanwhile, both hybrid aspen genotypes (174/10 and Wa13) showed huge drops in rooting ability, if affected by *P. tremulae* (decrease of 49–100%). Still, these two genotypes differed from each other in their responses to the first-stage bacterial inoculation. In 174/10, the first stage treatment with *Pseudomonas* sp. slightly mitigated the negative effect of *P. tremulae* (decrease of 49%). In contrast, *Pseudomonas* sp. had its own strong negative effect on the culture of genotype Wa13 (decrease of 62%) and the combination of *Pseudomonas* sp. and *P. tremulae* resulted in the total absence of rooted explants for this genotype.

### 3.3. Effect of Bacterial Inoculation on Populus Shoot Growth and Leaf Biochemistry

**Figure 4:**
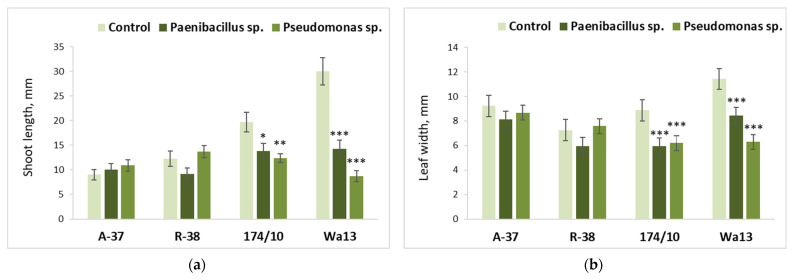
Average shoot length (**a**) and leaf width (**b**) of different *Populus* genotypes (A-37 and R-38, *P. tremula*; 174/10 and Wa13, *P. tremula* × *P. tremuloides*), inoculated with *Paenibacillus* sp. or *Pseudomonas* sp. bacteria. Statistically significant differences from the bacterium-free control variant are indicated (for each genotype separately): * *p* < 0.05; ** *p* < 0.01; *** *p* < 0.001.

Direct effect of nutrient medium inoculation with *Paenibacillus* sp. or *Pseudomonas* sp. bacteria on shoot development of different *Populus* genotypes is shown in Figure 4. In both aspen genotypes (A-37 and R-38), neither average shoot length (Figure 4a), nor leaf width (Figure 4b) was affected by either of the studied bacteria. A different situation was observed for both hybrid aspen genotypes (174/10 and Wa13), the shoot development parameters of which were significantly decreased by both *Paenibacillus* sp. and *Pseudomonas* sp. Of those two genotypes, Wa13 was particularly negatively affected, in terms of average shoot length, by *Paenibacilllus* sp. and, even more so, by *Pseudomonas* sp. (Figure 4a).

**Figure 5:**
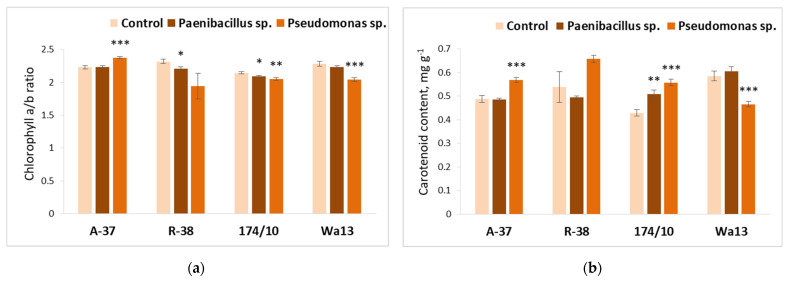
Average chlorophyll a/b ratio (**a**) and carotenoid content (**b**) in the leaves of different *Populus* genotypes (A-37 and R-38, *P. tremula*; 174/10 and Wa13, *P. tremula* × *P. tremuloides*), inoculated with *Paenibacillus* sp. and *Pseudomonas* sp. bacteria. Statistically significant differences from the bacterium-free control variant are indicated (for each genotype separately): * *p* < 0.05; ** *p* < 0.01; *** *p* < 0.001.

Figure 5 illustrates bacterial effects on the pigment contents in *Populus* leaves. The obtained results show that treatment with bacterium *Pseudomonas* sp. significantly increased chlorophyll a/b ratio in the A-37 genotype (Figure 5a). Meanwhile, a different situation was observed for both hybrid aspens, whose chlorophyll a/b ratio was significantly decreased in response to this bacterium (Figure 5a).

Similar effects of bacterium *Pseudomonas* sp., as induced on chlorophyll a/b ratio, were also found the carotenoid content (Figure 5b) in aspen genotype A-37 and in hybrid aspen genotype Wa13, with the first genotype showing a positive and the latter showing a negative response. Additionally, in respect to carotenoid content, the response of hybrid aspen genotype 174/10 to *Pseudomonas* sp. was quite similar to that of A-37 and different from Wa13.

*Paenibacillus* sp. had a negative effect on the chlorophyll a/b ratio in genotypes R-38 and 174/10; however, inoculation with this bacterium caused a significant positive increase in the carotenoid content of genotype 174/10.

**Figure 6:**
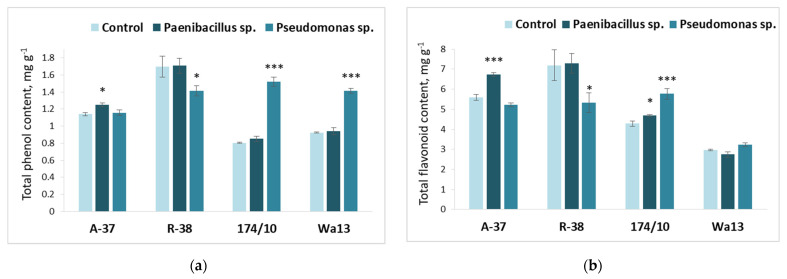
Average total phenol (**a**) and flavonoid (**b**) content in the leaves of different *Populus* genotypes (A-37 and R-38, *P. tremula*; 174/10 and Wa13, *P. tremula* × *P. tremuloides*), inoculated with *Paenibacillus* sp. and *Pseudomonas* sp. bacteria. Statistically significant differences from the bacterium-free control variant are indicated (for each genotype separately): * *p* < 0.05; *** *p* < 0.001.

The total phenolic content (Figure 6a) highly increased in response to bacterium *Pseudomonas* sp. in both hybrid aspen genotypes. Meanwhile, the same bacterium decreased the phenolic content in aspen genotype R-38. *Paenibacillus* sp. had a slight positive effect on total phenol content in genotype A-37.

The results presented in Figure 6b reveal obvious differences in total flavonoid content among the studied genotypes. Genotype A-37 showed a positive response to bacterium *Peanibacilus* sp. and genotype R-38 showed negative response to bacterium *Pseudomonas* sp. Genotype 174/10 showed a positive response to both *Paenibacillus* sp. and *Pseudomonas* sp.

### 3.4. Long-Term Effect of the Studied Bacteria on Populus Shoot Growth and Leaf Biochemistry after Inoculation with Phellinus tremulae

**Figure 7:**
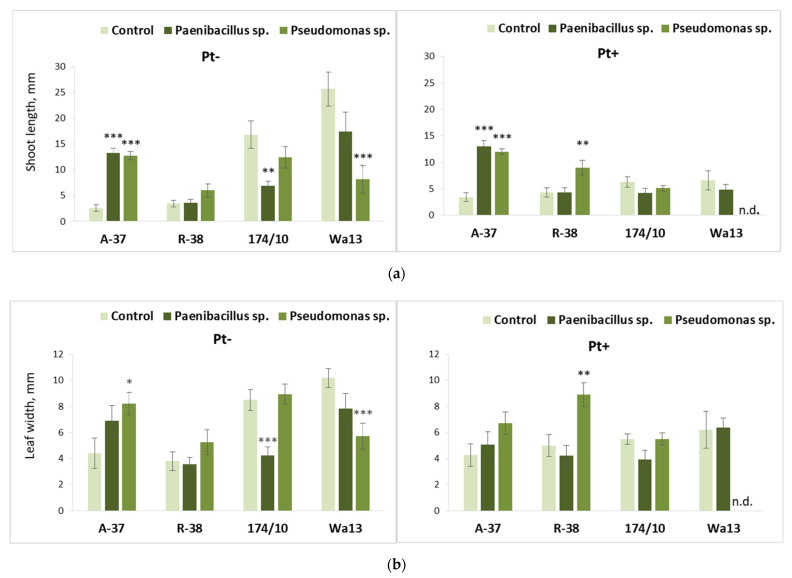
Average shoot length (**a**) and leaf width (**b**) of different *Populus* genotypes (A-37 and R-38, *P. tremula*; 174/10 and Wa13, *P. tremula* × *P. tremuloides*), whose explants were first cultured on the nutrient medium inoculated with *Paenibacillus* sp. or *Pseudomonas* sp. bacteria, then transferred onto fresh nutrient medium, either sterile (‘Pt−’) or inoculated with fungus *Phellinus tremulae* (‘Pt+’). Statistically significant differences from the control variant, which was not treated with bacteria during the first culture stage, are indicated (for each genotype separately): * *p* < 0.05; ** *p* < 0.01; *** *p* < 0.001. ‘N.d.’ means that, because of decreased explant viability, no reliable data were obtained for a particular variant.

The long-term effect of *Paenibacillus* sp. or *Pseudomonas* sp. bacteria on shoot development of different *Populus* genotypes is shown in Figure 7. These results were obtained after the explants from the bacterium-inoculated nutrient medium were transferred and cultured on either sterile or fungus-inoculated medium (second culture stage). The difference between the two *Populus* genotype pairs (aspens A-37 and R-38 and hybrid aspens 174/10 and Wa13) in their responses to the studied microorganisms remained distinct during the second experimental stage. Investigated aspen genotypes, in contrast to the hybrids, continued to show a generally positive response; however, the two aspen genotypes still differed from each other in some respects.

It was found that both *Paenibacillus* sp. and *Pseudomonas* sp. caused huge increases in the average shoot length of genotype A-37 during the second culture stage, irrespectively of the *P. tremulae* inoculation (Figure 7a). Although the bacterial effect on the leaf development of A-37 explants was not so clear, *Pseudomonas* sp. still caused a significant leaf width increase in this genotype on the sterile second-stage medium (Figure 7b). A somewhat different situation was observed in aspen genotype R-38, where bacterial effect on shoot development was not significant, if explants, after bacterial treatment, were transferred onto sterile nutrient medium; however, *Pseudomonas* sp. approximately doubled both the average shoot length (Figure 7a) and leaf width (Figure 7b) of R-38 explants (108% and 78% respectively) on the nutrient medium inoculated with *P. tremulae*. The hybrid aspen genotypes were generally negatively affected by the microorganisms. It can be noted that a significant decrease of average shoot length was caused by *Paenibacillus* sp. in genotype 174/10 and by *Pseudomonas* sp. in genotype Wa13 (Figure 7a). The same pattern of bacterial effects on the two hybrid aspen genotypes was observed regarding leaf width (Figure 7b). However, these negative bacterial effects on the hybrid aspens were identifiable only on the sterile second stage medium; meanwhile, on the medium inoculated with *P. tremulae*, bacterial effects on the shoot development parameters were insignificant because the fungus itself prevented shoot development in both hybrid genotypes.

### 3.5. Long-Term Bacterial Inoculation Effect on Populus Leaf Biochemistry after Inoculation with P. tremulae

**Figure 8:**
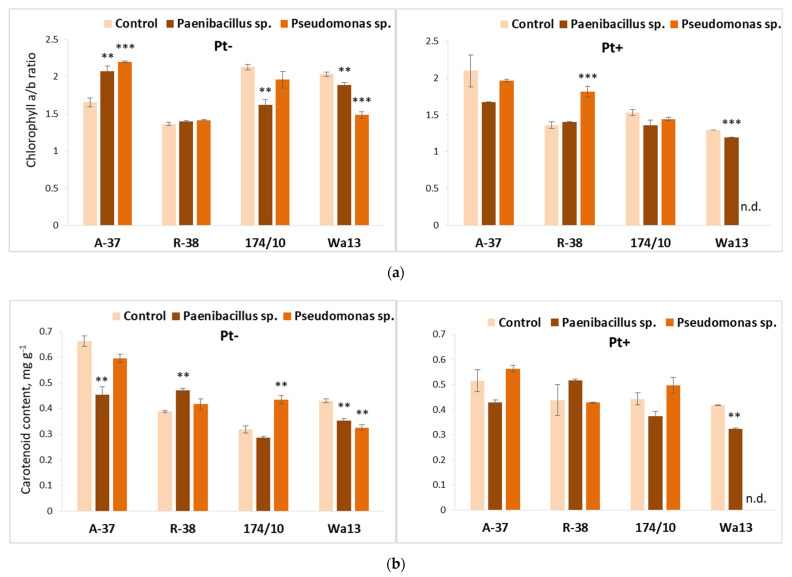
Average chlorophyll a/b ratio (**a**) and carotenoid content (**b**) in the leaves of different *Populus* genotypes (A-37 and R-38, *P. tremula*; 174/10 and Wa13, *P. tremula* × *P. tremuloides*), whose explants were first cultured on the nutrient medium inoculated with *Paenibacillus* sp. or *Pseudomonas* sp. bacteria, then transferred onto fresh nutrient medium, either sterile (‘Pt−’) or inoculated with fungus *Phellinus tremulae* (‘Pt+’). Statistically significant differences from the control variant, which was not treated with bacteria during the first culture stage, are indicated (for each genotype separately): ** *p* < 0.01; *** *p* < 0.001. ‘N.d.’ means that, because of decreased explant viability, no reliable data were obtained for a particular variant.

The long-term bacterial effects on the photosynthesis pigment (chlorophyll and carotenoid) content in *Populus* leaves are shown in Figure 8.

The chlorophyll a/b ratio had the smallest value in genotype R-38 and this parameter remained unresponsive to previous bacterial treatment if a fungus-free medium was used for the second stage (Figure 8a). However, genotype R-38 showed a positive response to the previous *Pseudomonas* sp. treatment (increase of 33%), if the second culture stage was conducted on the medium inoculated with *P. tremulae*. Long-term effects on chlorophyll a/b ratio of both *Paenibacillus* sp. and *Pseudomonas* sp. inoculation were significant in fungus-free aspen genotype A-37 and in hybrid aspen genotype Wa13; the response of A-37 to both bacteria was positive and the response of Wa13 to the same was negative (Figure 8a). Additionally, genotype Wa13 was negatively affected by *P. tremulae*, if, in the first stage, the explants were inoculated with *Paenibacillus* sp. Moreover, *Paenibacillus* sp. had a negative effect on hybrid 174/10 in this respect.

Long-term bacterial effects on carotenoid content were best seen if the medium of the second stage was not inoculated with *P. tremulae* (Figure 8b). It was noted that the long-term effect of *Paenibacillus* sp. decreased carotenoid content in genotypes A-37 and Wa13, but increased it in genotype R-38; meanwhile, *Pseudomonas* sp. decreased carotenoid content in genotype Wa13, but increased it in genotype 174/10. Only *Paenibacillus* sp. had any sort of significant effect on carotenoid content in *P. tremulae*-inoculated shoots; it was negative in genotype Wa13.

**Figure 9:**
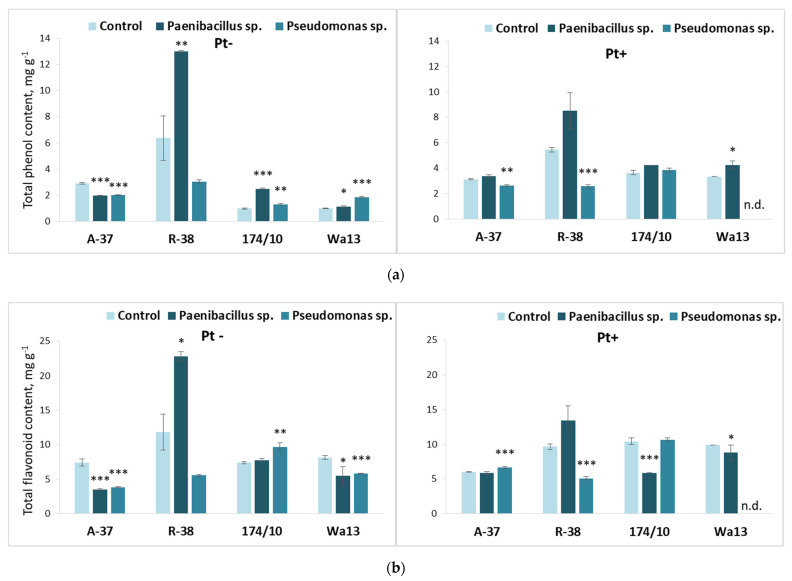
Average total phenol (**a**) and flavonoid (**b**) content in the leaves of different *Populus* genotypes (A-37 and R-38, *P. tremula*; 174/10 and Wa13, *P. tremula* × *P. tremuloides*), whose explants were first cultured on the nutrient medium inoculated with *Paenibacillus* sp. or *Pseudomonas* sp. bacteria, then transferred onto fresh nutrient medium, either sterile (‘Pt−’) or inoculated with fungus *Phellinus tremulae* (‘Pt+’). Statistically significant differences from the control variant, which was not treated with bacteria during the first culture stage, are indicated (for each genotype separately): * *p* < 0.05; ** *p* < 0.01; *** *p* < 0.001. ‘N.d.’ means that, because of decreased explant viability, no reliable data were obtained for a particular variant.

The long-term bacterial effects on the phenol and flavonoid content in *Populus* leaves are shown in Figure 9.

Particularly interesting results were obtained in the case of aspen genotype R-38; here, the previous treatment with bacterium *Peanibacillus* sp. led to increased concentrations of stress-resilience indicators, including not only previously described carotenoids (Figure 8b) but also phenolic and flavonoid compounds (increase of 105% and 93%, respectively) (Figure 9). In both instances, this was observed in fungus-free media.

In fungus-free genotype A-37, total phenol content diminished irrespective of bacteria used; however, similarly, it slightly increased in both hybrid aspen genotypes, irrespective of bacterial inoculation. When inoculated with *P. tremulae*, *Pseudomonas* sp. inoculated shoots had lower total phenol content than their respective controls in both aspen genotypes and, in genotype Wa13 previously inoculated with *Paenibacillus* sp., total phenol content slightly increased.

Both bacteria decreased flavonoid content in genotypes A-37 and Wa13, while *Pseudomonas* sp. increased it in genotype 174/10 in fungus-free media. Apart from a slight increase in flavonoid content in *P. tremulae*-inoculated A-37, post *Pseudomonas* sp. inoculation, *Paenibacillus* sp. had a significant negative impact on both hybrid aspens, while *Pseudomonas* sp. had a negative impact on the flavonoid content of R-38.

## 4. Discussion

Different bacteria have been successfully studied in attempts to control various plant pathogens and promote plant growth [46,47,48,49,50,51]. Both *Pseudomonas* and *Paenibacillus* species have been shown to have positive effects on plant growth in previous studies; this is sometimes linked with nitrogen fixation or phytohormone production [9,48,52], etc. In previous studies, *Paenibacillus* sp. were shown to have a positive effect not only on *Populus* root-system growth in vitro [9,53], but also on other trees [54,55,56,57]. Additionally, both bacterial genera have attracted considerable interest because of their ability to degrade chitin, a key compound found in the walls of fungal cells [46,58].

If compared to *Paenibacillus*, the data about positive effects of *Pseudomonas* bacteria on trees are less abundant. Still, it was reported that *P. fluorescens*, isolated from apple trees, induced a positive effect on apple shoots in vitro [59]; *P. fulva* was also shown to have a positive effect on pine seedling growth [51] and *P. frederiksbergensis* increased *Populus euramericana* growth in pot trials [60]. The present study showed that bacteria from the genera *Paenibacillus* and *Pseudomonas* can be applied for the growth promotion of in vitro-propagated *Populus* plants, but the particular outcome largely depends on a specific genotype and was temporally variable, with the enhancements becoming statistically significant in the second-stage trials (after 3 months) and not after the first stage (after 6 weeks).

To have a more complete view about the microorganism influence on different *Populus* genotypes, not only growth parameters but also biochemical changes should be considered. During this study, both tested bacteria were shown to have an impact on chlorophyll a/b ratio and carotenoid content, with *Pseudomonas* sp. having a more notable effect in both respects. However, this too seems to be genotype dependent, as *Pseudomonas* sp. mostly decreased chlorophyll a/b ratio in tested hybrids but had a largely positive effect in one of the *P. tremula* genotypes. Our results indicate that higher chlorophyll a/b ratio and carotenoid concentration was related to better rooting in genotype A-37, inoculated with *Pseudomonas* sp., in the first-stage trials. The same trend was notable in the second stage, whereupon the same genotype produced more chlorophyll in response to the bacterium. Additionally, shoot growth and leaf width of this genotype were positively affected. Otherwise, the effect of both species on carotenoid content was fluctuating.

Carotenoids, as well as chlorophylls, are photosynthesis pigments; however, their functionality in plants is somewhat different [61]. While they both participate in photosynthesis, increases in chlorophyll content are usually linked with the enhancement of plant viability [57,62] (even linking it to bacterial nitrogen fixation or siderophore production in bacteria–plant interaction studies [63,64]), while carotenoids are linked with plant stress, antioxidative activity and phytohormone production [65,66,67,68,69]. Several endophytes, both fungal and bacterial, have been shown to have an impact on plants’ carotenoid [61,62,65,70] and chlorophyll content [57,62,71]. Increases in both, ultimately lead to enhancements in net photosynthesis and, thus, carbohydrate synthesis, which, in turn, benefits the plant [65]. Additionally, Benyas et al. noted, that variations in chlorophyll synthesis are closely related to plants’ stress response [72].

The present study highlights the effects tested microorganisms may have on *Populus* SM synthesis. In previous studies, SMs, such as phenols and flavonoids, have been linked with various functions in plants: response to environmental stress, resistance to pathogens and mediation of interactions [14,16,17,18,73,74,75,76]. Flavonoids specifically, are known to be associated with *Populus* sp. resistance to pathogens [77]. Moreover, plant-associated microorganisms have also been linked with these processes, usually in a regulatory role [16]. It is widely considered that higher production of SMs is associated with enhanced chemical defensive response [18]. This leads to the use of a considerable number of elicitors of biotic and abiotic origin in improving production of the SMs in plant cells [74,78,79].

In our study, *Paenibacillus* sp. increased total phenol and flavonoid content in genotype A-37 and *Pseudomonas* sp. increased total phenols in both hybrid aspen during the first stage and both stages, respectively. However, most notably, during the second-stage trials, *Paenibacillus* sp. increased total phenol content in *P. tremula* genotype R-38 by 105%. However, this was not linked with a significant growth increase. The same trend was obvious in total flavonoid content, with it being increased by 93%. This variation is not unexpected, as SMs are affected not only by the plant genotype, but also by the physiological and developmental stage and stress levels of said plant [16,18,73].

Cesco et al. indicated that flavonoids can serve to enhance or weaken plant growth by mediating plant–bacterial interactions [80]. Various microbes have been shown to have an effect on plant SM content. For example, the positive effects of bacterium *Pseudomonas* sp. were described by Nowak [81] and Grover et al. [82]. Authors indicated that bacterium *Pseudomonas* sp. promoted increased synthesis of phenolic compounds and chlorophylls and promoted the growth of plant roots. Furthermore, it was demonstrated that orchid endophytic fungus could both promote its growth and enhance its flavonoid content [83]. Studies on *Paenibacillus* spp.’s effects on the SM concentration in plants are scarse, however.

Pathogenic fungus *P. tremulae* had a varied effect on plant growth, pigment content and SM production. Hybrid aspens were especially negatively impacted by the fungus, with loss of viability in genotype Wa13, which, in untreated control, had relatively low concentration of SMs. This correlates with the fact that *P. tremulae* is known to have a more adverse effect on *P. tremuloides* [34], from which both tested hybrids are descended. Additionally, hybrid aspens responded with decreased viability not only to the fungus, but also to the bacterial treatments. This demonstrates that bacteria may affect different genotypes differently, as is noted in other studies [9,54,55], as well as becoming opportunistic pathogens [84].

Interestingly, despite the pathogenic nature of *P. tremulae*, Eurasian aspen genotype R-38 was positively affected by it in concurrence with previous *Pseudomonas* sp. inoculation, with increases in rooting, shoot length, leaf width and chlorophyll a/b ratio by 129%, 108%, 78% and 33%, respectively. Moreover, the fungus alone had a positive effect on rooting, up to 120% in the same genotype. This can be linked with the impact *Pseudomonas* sp. had on total phenol and flavonoid contents in both *P. tremula* genotypes; thus, our results indicate that the changes in SM concentration in studied genotypes was potentially related to their antipathogenic response, even though this was not linear.

Previous studies show that *Pseudomonas* are known to increase plant resistance to stress and pathogens [51,85,86,87]; however, to our knowledge, no studies on trees or with *P. tremulae* have been conducted thus far, though several older studies demonstrated the biocontrol capabilities of fungi against this pathogen [14,22,23,35].

While, additionally to the present results, other studies have also found that increases in microbe-stimulated plant SM production are linked with growth promotion and pathogen resistance, this link is often complex and hard to interpret, as the relationship between plants and their microbiota is a dynamic one—studies have shown that microbes can affect SMs that, in turn, modulate plants’ microbiome [16].

## 5. Conclusions

The studied microorganisms, bacteria *Paenibacillus* sp. and *Pseudomonas* sp. and fungus *Phellinus tremulae*, had significant effects on growth parameters, photosynthetic pigment content and secondary metabolite production in four different *Populus* genotypes, cultured in vitro. However, these effects were variable and clearly genotype-dependent. It was found that the two genotypes (both *Populus tremula* × *P. tremuloides*), whose development in vitro was significantly damaged by *Phellinus tremulae*, were also characterized by certain responses to the studied bacteria: decreased shoot development in response to both *Paenibacillus* sp. and *Pseudomonas* sp. and increased phenol content in response to *Pseudomonas* sp. In turn, these responses were lacking in the other two tested genotypes (both *Populus tremula*). Additionally, these genotypes showed in vitro resistance to the fungus and positive, long-term growth responses to bacterial inoculation, even synergistic with the subsequent fungal inoculation. Hence, the studied bacteria were demonstrated as a potential tool both for the detection of fungus-susceptible *Populus* genotypes and for the improved of in vitro propagation of fungus-resistant genotypes.

Based on the results of these preliminary findings, future studies will be geared towards investigating how varied concentrations of bacteria may affect the plants and *P. tremulae*. Subsequent ex vitro trials are also to begin.

## Figures and Tables

**Table 1:** Scheme of the inoculation experiments, indicating inoculation of plant nutrient medium with different microorganisms (bacteria that were determined to be *Paenibacillus* sp. and *Pseudomonas* sp. and fungus, *Phellinus tremulae*) during two culture stages.

Experimental Variant	First Culture Stage (Six Weeks)	Second Culture Stage (Six Weeks)
Control	-	-
+Pt.	-	*P. tremulae*
Paen	*Paenibacillus* sp.	-
Paen + Pt	*Paenibacillus* sp.	*P. tremulae*
Pseu	*Pseudomonas* sp.	-
Pseu + Pt	*Pseudomonas* sp.	*P. tremulae*

**Table 2:** Percentage of rooted explants in the in vitro cultures of four *Populus* genotypes after second stage of culturing with different microorganisms. In the first stage, the nutrient medium was inoculated with either *Paenibacillus* sp. (‘Paen’) or *Pseudomonas* sp. (‘Pseu’) bacteria. For the second stage, explants from each variant were transferred onto fresh medium, either sterile or inoculated with fungus *P. tremulae* (‘+ Pt’). The control group of explants was cultured on sterile media during both stages.

Experimental Variant	Aspen (*P. tremula*)		Hybrid Aspen (*P. tremula* × *P. tremuloides*)	
	A-37 ^1^	R-38	174/10	Wa13
Control	41.67 ± 14.86	33.33 ± 12.6	80 ± 9.18	87.5 ± 8.54
+Pt	21.43 ± 11.38	73.33 ± 11.82 *	25 ± 9.93 ***	12.5 ± 8.54 ***
Paen	61.54 ± 14.04	37.5 ± 12.5	52.94 ± 12.48	58.82 ± 12.3
Paen + Pt	28.57 ± 12.53	31.25 ± 11.97	23.53 ± 10.6 ***	16.67 ± 9.04 ***
Pseu	85.71 ± 9.71 *	50 ± 12.91	76.47 ± 10.6	33.33 ± 11.43 ***
Pseu + Pt	57.14 ± 13.73	76.47 ± 10.6 *	41.18 ± 12.3 *	0 ***

^1^ Statistically significant difference from the control variant (for each genotype separately) are indicated: * *p* < 0.05; *** *p* < 0.001.

## Data Availability

Complete study data is available upon request.
